# Treatment of advanced thyroid cancer refractory to therapy

**DOI:** 10.1186/1756-6614-6-S2-A22

**Published:** 2013-04-05

**Authors:** Barbara Jarząb

**Affiliations:** 1 Department of Nuclear Medicine and Endocrine Oncology, Maria Sklodowska-Curie Memorial Cancer Centre and Institute of Oncology, Gliwice Branch, Poland

## 

Radioiodine treatment constitutes the most effective therapeutic option of advanced differentiated thyroid cancer. Unfortunately, about 30% cancers do not show radioiodine uptake or do not respond to therapy.

Thyrosine kinase inhibitors (TKI), among them axitinib, cabozantinib, lenvatinib, motesanib, pazopanib, sorafenib, sunitynib and vandetanib, constitute a new group of drugs implemented to therapy of both differentiated thyroid cancer (DTC) and medullary thyroid cancer (MTC). They inhibit growth factor receptors which play crucial role in processes of growth, differentiation and maturation of neoplastic cell. Detailed information related to mechanism of action of each drug as well as to conducted clinical trials are given in the table below:

**Table 1:** 

Drug name	Mechanism of action	Clinical trials (phase)	Indications
MOTESANIB	VEGFR1,2,3, PDGFR, c-KIT, RET	II	MTC, DTC
SORAFENIB	B-RAF, VEGFR1, VEGFR2	II/III	MTC
AXITINIB	VEGFR, c-KIT, PDGFR-B	II	DTC
SUNITINIB	VEGFR1, 2, PDGFR, c-KIT, FLT3, RET	II	DTC
LENVATINIB	VEGFR1,2,3, FGFR1 PDGFR	II/III	MTC
CABOZANTINIB	MET, VEGFR2, RET	III	DTC
PAZOPANIB	VEGFR, PDGFR, c-KIT	II	MTC
VANDETANIB	RET, VEGFR, VEGFR2, EGFR	II	DTC

Until now the only registered drug in advanced MTC is vandetanib. Its efficacy has been proved in phase III study. Significant prolongation of progression free survival (PFS) was observed for patients receiving vandetanib compared with placebo group (30.5 months *vs*. 19 months, respectively). Published results of phase II studies have preliminarily proved the efficacy of axitinib, sorafenib and sunitinib in DTC (beneficial therapeutic effects of partial regression or disease stabilization was noticed in 68%, 76% and 75% cases, respectively). The highest response rate (partial regression) was obtained in DTC patients treated with pazopanib (49%). Whereas, in phase II clinical trials with cabozantynib, motesanib and sorafenib carried out in MTC, disease control was achieved in 90%, 83% and 94% patients, respectively.

The most common side effects are skin reactions such as photosensitivity, rash, hand-food syndrome, arterial hypertension, gastrointestinal – diarrhea, nausea, vomiting, stomatitis and decrease in body weight. Majority of them have slight or moderate intensiveness (G1 and G2 according to Common Terminology Criteria for Adverse Events). The tolerability of TKI is acceptable and does not affect the quality of life.

